# Expression of Concern: Silencing of SOX12 by shRNA suppresses migration, invasion and proliferation of breast cancer cells

**DOI:** 10.1042/BSR-2016-0053_EOC

**Published:** 2022-11-16

**Keywords:** breast cancer, cell cycle arrest, invasion, migration, proliferation, SOX12

The Editorial Office has been made aware of potential issues surrounding the scientific validity of this paper, hence has issued an expression of concern to notify readers whilst the Editorial Office investigates. Some of the western blot bands for Figure 5C, specifically the Cyclin/BT474 bands, appear to be highly similar to the Figure 5C B-Carwnin/HO-8910 bands in a paper by Cheng et al. 2015 (doi: 10.18632/oncotarget.4541), which has no related authors.

